# C1431T Variant of PPARγ Is Associated with Preeclampsia in Pregnant Women

**DOI:** 10.3390/life11101052

**Published:** 2021-10-07

**Authors:** Fulin Liu, Christine Rouault, Karine Clément, Wencan Zhu, Séverine A. Degrelle, Marie-Aline Charles, Barbara Heude, Thierry Fournier

**Affiliations:** 1 Pathophysiology & Pharmacotoxicology of the Human Placenta, Pre & Postnatal Microbiota, 3PHM, INSERM, Université de Paris, F-75006 Paris, France; fulin.liu@etu.u-paris.fr (F.L.); severine.degrelle@inserm.fr (S.A.D.); 2 Nutrition et Obesités: Approches Systémiques Research Unit, INSERM, Sorbonne Université, F-75013 Paris, France; christine.rouault@nutriomique.org (C.R.); karine.clement@psl.aphp.fr (K.C.); 3 Nutrition Department, Pitié-Salpêtrière Hospital, Assistance Publique Hôpitaux de Paris, F-75013 Paris, France; 4 UMR MIA-Paris, INRAE, AgroParisTech, Université Paris-Saclay, F-75005 Paris, France; wencan.zhu@agroparistech.fr; 5 Inovarion, F-75005 Paris, France; 6 Centre for Research in Epidemiology and Statistics (CRESS), INSERM, INRAE, Université de Paris, F-75004 Paris, France; marie-aline.charles@inserm.fr; 7 Ined, Unité Mixte Inserm-Ined-EFS ELFE, F-75020 Paris, France

**Keywords:** PPARγ, SNPs, machine learning, models, preeclampsia

## Abstract

Peroxisome proliferator-activated receptor γ (PPARγ) is essential for placental development, whose SNPs have shown increased susceptibility to pregnancy-related diseases, such as preeclampsia. Our aim was to investigate the association between preeclampsia and three PPARγ SNPs (Pro12Ala, C1431T, and C681G), which together with nine clinical factors were used to build a pragmatic model for preeclampsia prediction. Data were collected from 1648 women from the EDEN cohort, of which 35 women had preeclamptic pregnancies, and the remaining 1613 women had normal pregnancies. Univariate analysis comparing preeclamptic patients to the control resulted in the SNP C1431T being the only factor significantly associated with preeclampsia (*p* < 0.05), with a confidence interval of 95% and odds ratio ranging from 4.90 to 8.75. On the other hand, three methods of multivariate feature selection highlighted seven features that could be potential predictors of preeclampsia: maternal C1431T and C681G variants, obesity, body mass index, number of pregnancies, primiparity, cigarette use, and education. These seven features were further used as input into eight different machine-learning algorithms to create predictive models, whose performances were evaluated based on metrics of accuracy and the area under the receiver operating characteristic curve (AUC). The boost tree-based model performed the best, with respective accuracy and AUC values of 0.971 ± 0.002 and 0.991 ± 0.001 in the training set and 0.951 and 0.701 in the testing set. A flowchart based on the boost tree model was constructed to depict the procedure for preeclampsia prediction. This final decision tree showed that the C1431T variant of PPARγ is significantly associated with susceptibility to preeclampsia. We believe that this final decision tree could be applied in the clinical prediction of preeclampsia in the very early stages of pregnancy.

## 1. Introduction

Preeclampsia, which is characterized by high blood pressure and concurrent proteinuria, is a complication of pregnancy that usually manifests after 20–25 weeks of pregnancy [1]. This disease is highly associated with morbidity and mortality for both the mother and the fetus because of its serious risks to fetal maturity and the maternal cardiovascular system [2]. Preeclampsia occurs in 5% to 7% of all pregnant women, leading to over 70,000 maternal deaths and 500,000 fetal deaths worldwide every year [3]. Between 2010 and 2016, an estimated 5.2% of pregnancies in France were affected by gestational hypertension, with 2% of pregnancies developing preeclampsia/eclampsia [4]. Many attempts have been made to accurately diagnose preeclampsia in the early stages. Typically, these are based on strategies such as analyses of metabolomic pathways and combined metabolomic/proteomic data [5,6,7]. A newly proposed method for the diagnosis of preeclampsia relies on combined detection of biomarkers such as sFLT1, sEng, and PlGF and performs well around 34 weeks of gestation [8,9,10]. This has achieved 89% predictive accuracy for preeclampsia in pregnancies before 32 gestational weeks, 75% in pregnancies before 37 weeks, and 47% in pregnancies after 37 weeks [11,12]. Despite the advances that have been made, there is still clearly room for improvement in achieving earlier and higher accuracy of prediction. For this reason, our goal was to develop a tool that could be used for earlier diagnosis of preeclampsia.

One novel diagnostic tool may be the use of genetic analysis. The protein product of a variant of the FLT1 gene, at locus rs4769613, has been identified as a pathogenetic factor for susceptibility to preeclampsia in pregnancy [13], as has locus rs9478812, located in an intronic region of the protein PLEKHGI [14]. Another relevant candidate could be peroxisome proliferator-activated receptor γ (PPARγ), of which multiple variants have been implicated in the development of numerous disorders. For example, variant C1431T has been associated with susceptibility to obesity in the European population [15,16,17], C161T (rs3856806) has been linked to the risk of essential hypertension and premature acute myocardial infarction [18,19,20], and the rs3856806 C–T substitution single nucleotide polymorphism (SNP) was found to increase the risk of colorectal cancer [21]. Moreover, previous work by our group has demonstrated an association between two variants of PPARγ (Pro12Ala and C1431T) and gestational diabetes [22]. Even though multiple studies have suggested that SNPs in various genes increase the risk of preeclampsia [23,24,25], the association between SNPs of PPARγ and the risk of preeclampsia has been poorly investigated.

PPARγ is a member of the nuclear hormone receptor subfamily that functions as a transcription factor by binding to target genes, many of which are involved in insulin sensitivity, metabolic processes such as adipogenesis and lipogenesis [26], and immunological processes such as inflammation and differentiation [27]. Complete knock-out of the PPARγ gene can lead to embryonic lethality [28], while milder deficiencies result in insufficient trophoblast differentiation and abnormal vasculogenesis in mice [29]. It has also been suggested that PPARγ plays a role in elevating blood pressure, proteinuria, endothelial dysfunction, and platelet aggregation, i.e., key features of preeclampsia in rats [30]. Multiple reports have linked variants of PPARγ with diseases such as coronary heart disease, cancer, metabolic syndrome, and especially obesity and diabetes [22,31,32,33,34]. The PPARγ gene is located in the human chromosome 3p25 and comprises nine exons. One of the most common structural SNPs is a proline (Pro) to alanine (Ala) substitution, Pro12Ala (rs1805192), which results from the mutation of cytosine to guanosine [18]. Compared with the normal Pro allele, the Ala-substituted allele leads to a reduction in activity of PPARγ [35], which can be a high-risk factor for the occurrence of obesity and type 2 diabetes [36,37]. In addition to the common Pro12Ala variant, the C1431T variant, located in exon six, has been associated with leptin concentrations [15] and body mass index [16,17], and the C681G variant was found to be linked with accelerated growth in young schoolchildren and increased adult height [38]. Given that SNPs in the PPARγ gene have been implicated in a wide range of diseases, we hypothesized that such variants may also play a role in preeclampsia.

**Figure 1:**
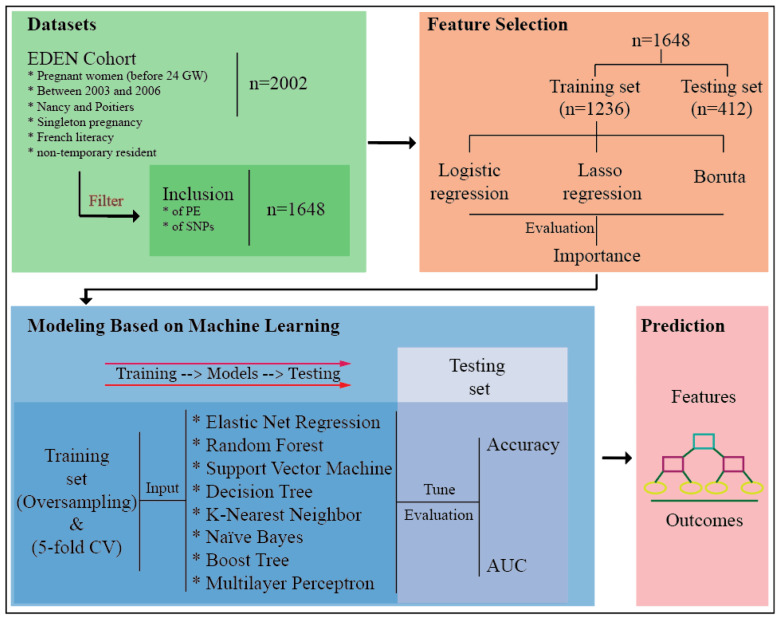
Schematic diagram of the study. Of the 2002 pregnant women in the EDEN mother–child cohort study, 1648 satisfied the inclusion and exclusion criteria and were recruited to this study. The dataset was randomly stratified into two parts, a training set and a testing set, according to a 3:1 ratio. Three methods were used to evaluate the importance of correlated features within the training set: logistic regression, lasso regression, and the Boruta algorithm. Eight machine-learning models were built, tuned, and trained on an oversampled training set with five-fold cross-validation (CV), followed by validation on the testing set. The performance of the models was evaluated using metrics of accuracy and the area under the receiver operating characteristic curve (AUC). The final model was used to build a decision tree for predicting preeclampsia. GW: gestational week; PE: preeclampsia; SNPs: single nucleotide polymorphisms.

In the current study, we aimed to investigate the association between the risk of preeclampsia and the Pro12Ala, C1431T, and C681G SNPs of PPARγ. Furthermore, we wanted to use the SNP data and clinical features from the national EDEN cohort study [39] to build a model for preeclampsia prediction. The conventional way to build a model is to apply generalized linear models, which are easy and fast to implement. However, erroneous specification of model parameters or assumptions can lead to bias in the results. We therefore applied new machine-learning methods that are able to fully consider complex relationships between the predictors and the outcome, with fine-scale argument tuning. In this way, potential bias can be, to some extent, diminished. A summary of the study procedure is shown in Figure 1.

## 2. Results

### 2.1. Overview of Maternal Clinical Features

**Figure 2:**
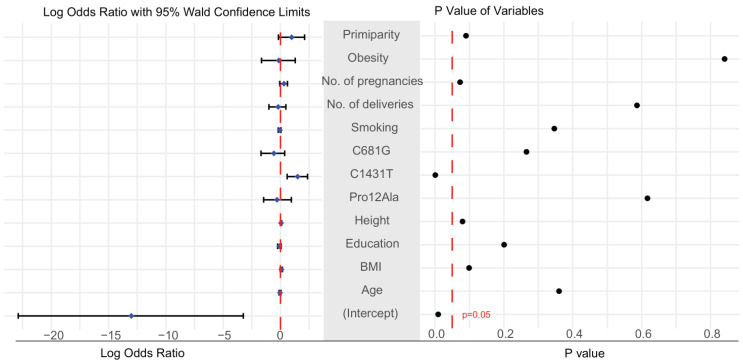
Evaluation of clinical features. The log odds ratios of all clinical features are presented on the left, with corresponding p-values on the right. Bars indicate the mean and 95% confidence interval of the log odds ratio. *P*-value less than 0.05 indicates significance. Age: maternal age at delivery; Height: maternal height; Education: maternal education; BMI: body mass index before pregnancy; Smoking: cigarette use.

Table 1 presents a summary of maternal clinical features from the control (n = 1613) and preeclampsia (n = 35) groups. According to t-test and chi-square tests, the only factor that was significantly different between the two groups was the presence in the maternal genome of the C1431T variant of PPARγ (Table 1). Similarly, logistic regression and analysis of the log odds ratio found that mothers carrying this variant had a higher risk of developing preeclampsia (*p*-value < 0.05; Figure 2). The 95% confidence interval of the odds ratio for maternal C1431T ranged from 4.90 to 8.75 (Supplementary Table S2). A comparison of the three genotype models confirmed that the maternal C1431T variant was the only factor associated with a significant difference in dominant and co-dominant models (Table 2). Clinical factors and genetic data were missing from 9.1% of the dataset (Supplementary Figure S1), while there was no significant change in the results before and after the imputation (Table S1). A summary of the data before and after imputation, showing no differences between the imputed and non-imputed summary tables, is shown in Table S1.

### 2.2. Selection of Candidate Features for the Prediction of Preeclampsia Using Three Methods: Boruta Algorithm, Lasso Regression, and Logistic Regression

**Figure 3:**
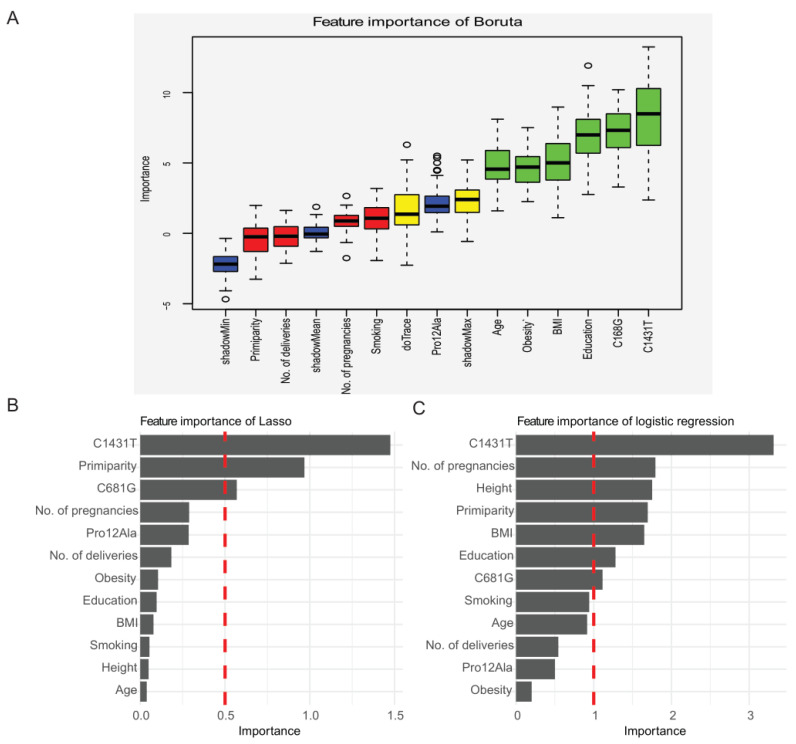
Feature selection. (**A**) Feature selection with the Boruta method. The Boruta algorithm is a wrapper built around the random forest classification algorithm, in which shadow features are generated from the shuffled values, which are duplicates of the dataset in each column. This leads to a range of values of the importance on features, in which blue boxplots depict the quantiles that indicate the weighted thresholds, based on Z-score (minimum, mean, and maximum), used for selecting features. Red boxplots represent features that were found to be unimportant, while green boxplots indicate important features. Yellow boxplots show features that may be important depending on the criteria used. (**B**) Feature selection with the lasso regression method, which minimizes the cost function to select those features of use and discards the useless or redundant features so that it can make its coefficient equal to 0. The weighted value can be calculated directly, with the soft threshold for importance as more than 0.5. (**C**) Feature selection with the logistic regression method based on a binary dependent variable model with the soft threshold for importance as more than 1. Age: maternal age at delivery; Height: maternal height; Education maternal education; BMI: body mass index before pregnancy; Smoking: cigarette use.

Three methods were used for feature selection: the Boruta algorithm, lasso regression, and logistic regression. The Boruta algorithm highlighted four features as important for predicting preeclampsia: education, maternal C681G and C1341T variants, and obesity. Relaxing the inclusion criteria resulted in inclusion of maternal delivery age, BMI, creating six important features (Figure 3A). Lasso regression singled out maternal C1341T and C681G variants and primiparity (Figure 3B), and logistic regression found maternal C1341T, number of pregnancies, primiparity, number of cigarettes, education, and BMI (Figure 3C). Therefore, the final features included in the model-building process were maternal C681G and C1341T, obesity, BMI, number of pregnancies, primiparity, number of cigarettes, and education.

### 2.3. Modeling Based on Machine Learning

The dataset was divided into a training set and a testing set. Due to the insufficiency of preeclampsia cases in the dataset, the training set was oversampled with respect to the incidence of preeclampsia, in order to obtain the balance between negative and positive cases as described in the Materials and Methods section. There were no differences between the datasets before and after oversampling in the representation of categorical factor and the mean and standard deviation of numeric factors (Supplementary Table S3). There was a wide degree of overlap between the distribution of positive and negative cases in the training set before and after balancing (Supplementary Figure S2D&E). The optimal combination of features was selected following a thorough process of tuning based on the optimal AUC of models (Supplementary Figure S6). 

**Figure 4:**
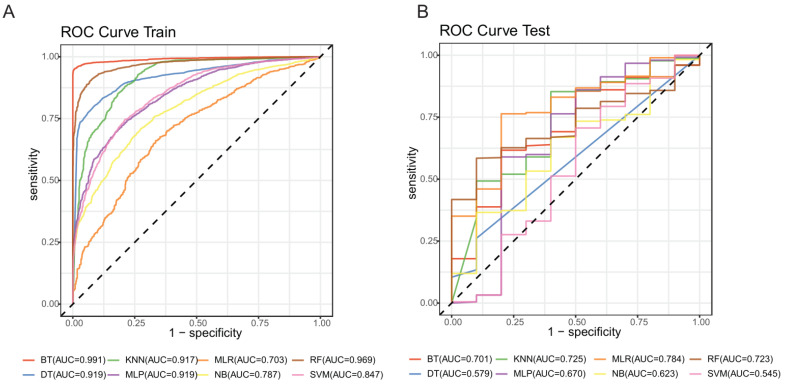
ROC curve of different algorithms. (**A**) ROC curves with the training set. (**B**) ROC curves with the testing set. AUC values are shown in the legends. AUC: area under the receiver operating characteristic curve; BT: boost tree; DT: decision tree; ENR: elastic net regression; KNN: k-nearest neighbor; MLP: multilayer perceptron; NB: naïve Bayes; RF: random forest; SVM: support vector machine; ROC: receiver operating characteristic.

The results of the final eight machine-learning models, with respect to model accuracy and AUC, are shown in Table 3 for both the training and testing sets. The optimal model was the boost tree model, for which the values for accuracy and AUC in the training set were 0.971 and 0.991, which was perhaps overfitted. We then validated the model in the testing set with the values for accuracy and AUC 0.951 and 0.701. The diagnostic performance of each of the machine-learning models (AUCs) is depicted in Figure 4.

### 2.4. Prediction Procedures of Boost Tree

**Figure 5:**
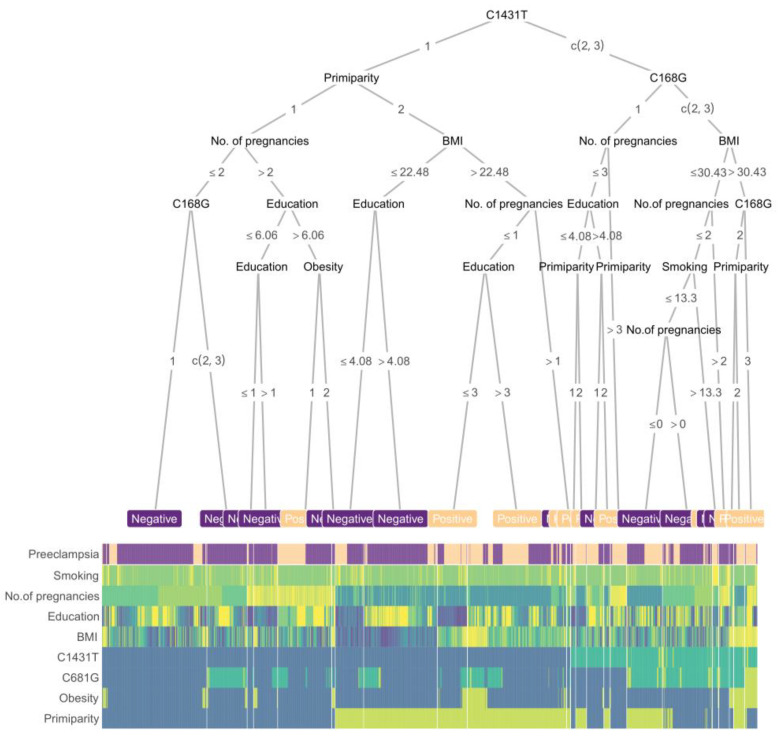
Boost tree and heatmap for predicting preeclampsia. The nodes of the boost tree represent the contributing features, while branches depict the threshold values. The first row of the heatmap presents the outcomes, while the lower rows present the predictor values. The colors represent the scaled value of a sample of each feature. Primiparity and maternal C681G function as the second nodes, followed by the number of pregnancies and BMI, while education played a less important role in the final decision. For genotypes, “1” represents no mutation in the allele, “2” means a single mutation, and “3” a double mutation; for primiparity and obesity, “1” means “no” while “2” means “yes”. The corresponding threshold values are shown on the branches. Integrated values of the clinical features of individuals are presented in the heatmap, corresponding to both positive and negative end outcomes. Age: maternal age at delivery; Height: maternal height; Education: maternal education; BMI: body mass index before pregnancy; Smoking: cigarette use.

A boost tree-based decision tree, along with a heatmap of scaled feature values, was constructed using the balanced training set (Figure 5). The clinical features that were determined to be important for prediction included the maternal PPARγ genotypes, primiparity, number of pregnancies, obesity, BMI, and education. As expected from the univariate tests, the maternal C1431T variant was the first key branching node of the tree. The simplicity of this procedure is intended to facilitate its possible use in clinical practice for the prediction of preeclampsia.

## 3. Discussion

In the present study, we identify a significant association between the C1431T SNP of PPARγ and preeclampsia. We also present a decision tree based on a boost tree model that represents a possible diagnostic procedure for pragmatic preeclampsia prediction, which could benefit early diagnosis regardless of gestational age.

The relationship between the PPARγ and preeclampsia has been widely reported. For example, the expression of PPARγ was increased in late onset preeclampsia, but not early onset preeclampsia, compared to normal pregnancy [40]; the activation of PPARγ by its agonist rosiglitazone can probably relieve the preeclampsia by reducing uterine perfusion pressure and benefiting placental vasculature [41,42]; PPARγ plays an important role in controlling endothelial function and blood pressure homeostasis, which is crucial for pathological preeclampsia [43]. However, a previous report states that there is no relationship between PPARγ polymorphism and the occurrence of preeclampsia [44]. In our study, the presence of the C1431T variant of PPARγ in the mother was shown to play a significant role in distinguishing between preeclamptic and normal pregnancies. However, no such role was detected for either the Pro12Ala or C681G SNPs. Interestingly, even though a chi-square test found no evidence of a link between C681G and preeclampsia, the inclusion of this variant in the final predictive model, as suggested by the feature selection process, was found to improve both the accuracy and AUC values of the machine-learning model.

Machine-learning algorithms are widely used to obtain better predictive accuracy compared to conventional generalized linear models in decision-making scenarios. Furthermore, they offer alternative strategies for the diagnosis of diseases based on clinical features [45,46,47,48]. Currently, there are eight machine-learning algorithms in wide use for modeling and building diagnostic procedures based on appropriate medical history: elastic net regression, random forest, support vector machine, decision tree, k-nearest neighbor, naïve Bayes, boost tree, and multilayer perceptron [49,50]. Several studies have compared different machine-learning methods for disease prediction under various clinical conditions, and the results have been mixed [51,52,53], suggesting that the optimal algorithm may vary depending on context. In our study, we applied and compared these eight machine-learning algorithms and addressed two common challenges in modeling: insufficiency and overfitting of the models. To prevent the former, we oversampled the positive cases to prevent inaccuracy due to the imbalance between positive and negative cases [54,55] and evaluated both the balanced and unbalanced versions of the training and testing sets. To avoid the latter, we used different approaches for preprocessing our dataset and applied five-fold CV to the training set, followed by validation on the testing set.

First, the original dataset was split into a training set and a testing set without balancing. In this case, the boost tree was the optimal model, with values of accuracy and AUC as 0.99 and 0.92 in the training set and 0.98 and 0.77 in the testing set, respectively (Table S4 and Figure S3). We then repeated this procedure, but first balanced the original dataset before splitting it into the training set and testing set. The boost tree remained the optimal method with accuracy and AUC values of 0.957 and 0.990 for the training set and 0.975 and 0.996 in the testing set, respectively (Table S5 and Figure S4). We suspected that overfitting may have influenced this model, owing to the internal relationship between the training set and the testing set that resulted from the data simulation. Lastly, we balanced the training set, only by oversampling the positive cases, and kept the testing set as it was after the split of the original dataset; those results are shown in Table 4 and Figure 4. Additionally, in the final model, we performed failure mode and effects analysis and calculated the F-score to verify the suitability of accuracy as a metric. We obtained high values for both training and testing sets (Table S6), which were generally in line with the accuracy values. However, the AUC value of the testing set in the final boost tree model was not high enough to be considered a convincing example; we hypothesize this might be due to the relatively small number of positive cases in the testing set. Further studies with larger datasets are needed to resolve this question.

In our study, the boost tree consistently yielded the highest accuracy and AUC value for both the training and testing sets, regardless of the methods used for preprocessing. For this reason, we used this approach to build a clinical flowchart for the evaluation of preeclampsia. This model outperformed both the screening methods currently recommended by the National Institute for Health and Care Excellence (a combination of maternal factors, uterine artery pulsatility index, mean arterial pressure, and PlGF; 41% accuracy) and ACOG guidelines (94% accuracy) [56], as well as methods based on biomarkers such as soluble fms-like tyrosine kinase 1 (sFlt1) and PIGF (77% accuracy) [57]. In addition, our model can be used to evaluate women before they become pregnant, as all of the predictors can be examined pre-pregnancy, and thus facilitates earlier diagnosis than existing alternatives [5,50,56,58,59]. However, despite the high degree of accuracy achieved here, the clear procedure for prediction, and the potential for earlier diagnosis, our model has some deficiencies that should be addressed. First, further studies in other regions or nations are needed, since patterns of SNPs can vary among populations and this can lead to inconsistent conclusions [60]. Second, certain clinical data were missing for some of our study subjects, and future studies on larger samples may be able to avoid this problem. Lastly, a larger number of positive cases should be included to balance the representation of preeclamptic and healthy pregnancies. Even though an appropriate algorithm was used to account for the imbalance here, it is possible that the difference between simulation and real cases may subtly influence the model performance.

## 4. Materials and Methods

### 4.1. Study Population

The EDEN study (study of pre-and post-natal determinants of children’s growth and development) is an ongoing mother–child cohort study that was set up in two locations in France, Nancy and Poitiers (France). A total of 2002 pregnant women were enrolled, on average at the 24th gestation week. More details about the EDEN study are available in [39]. The study received approval from the ethics committee (Comité Consultatif de Protection des Personnes dans la Recherche Biomédicale, N°02–70, 12 December 2002) of Kremlin Bicêtre Hospital and from CNIL (Commission Nationale Informatique et Liberté), the French data protection authority. Written informed consent was obtained twice from parents: at enrollment and again after the child’s birth. All research was performed in accordance with the relevant guidelines and regulations. Of the 2002 pregnancies, 1648 met the inclusion/exclusion criteria (Figure 1) to be included in the present work.

### 4.2. Clinical Features

At 24–28 gestational weeks, each mother was clinically examined and asked to complete a self-administered questionnaire. Clinical features, such as maternal weight and height, were measured during the examination, while data on personal history such as weight before pregnancy, educational level (from 1 to 10: 1 = none; 2,3,4 = tertiary; 5,6,7 = secondary; 8,9 = high school; 10 = other), and smoking habits were collected during an initial interview. Additional clinical features, such as gestational age at delivery and the number of previous pregnancies, were extracted from clinical records. Body mass index (BMI) was calculated according to the formula: BMI = kg/m^2^, where kg is the weight in kilograms and m^2^ is the height in meters squared. Values for continuous features are presented as mean ± S.D. and discrete factors are presented as percentages (N).

### 4.3. Genotyping

Maternal blood samples were collected during pregnancy and stored in −80°C freezers with alarm control. DNA was extracted from leukocytes using the QIAamp DNA Blood Mini Kit (QIAGEN) according to the manufacturer’s instructions. Genotyping of the SNPs was conducted using one of two techniques. For the first 729 women enrolled in the study, a LightCycler apparatus (Roche Diagnostics, Meylan, France) and hybridization probes were used, with primers and probes designed and synthesized by TIB MOLBIOL (Berlin, Germany). The PCR mixture (10 µl total volume) contained 20 ng of DNA, 1X Fast Start DNA master hybridization probes, 0.5 µM of primers, 0.15 µM of probes, and 3 mM MgCl_2_. Melting curve analysis was applied to monitor SNPs genotyping. For the remaining women (1024), the TaqMan procedure (Applied Biosystems, Foster City, CA, USA) was used with similar reagent preparation, as described above. DNA samples were amplified by PCR on a 96-well plate with the following cycling parameters: denaturation at 95°C for 10 min, and 40 cycles of 92°C for 15 s and 60°C for 1min. The results of the TaqMan assays were read on a 7900HT Fast Real-Time PCR System (Applied Biosystems, Foster City, CA, USA), and alleles were called using SDS software (Applied Biosystems, Foster City, CA, USA). The genotyping call rate of the three SNPs was above 98% in each case, including with the duplicate controls. Further details on the genotyping primers, probes, and PCR conditions are available from the corresponding author.

### 4.4. Basic Statistical Analyses

Basic statistical analyses were performed based on R software (version 4.0.4) with basic packages in Rstudio (PBC, Boston, MA, USA, http://www.rstudio.com/), an integrated development environment for R. Maternal clinical features were described separately in women with and without preeclampsia. The distribution of missing data was visualized using the R package *mice* (version 3.11) [61], and imputation of missing data was carried out using the R package *Imputation* and visualized using *missForest* (version 1.4) [62]. General view of features and individuals in the dataset was presented using principal component analysis using *FactoMineR* (version 2.3) and visualized by *factoextra* (version 1.0.7), taking three components into consideration [63]. Student’s t-test was used to compare continuous features between groups, and a chi-square test was used for comparing discrete features. Fisher’s exact test was applied when any of the cell values of a contingency table were less than five. Multivariate logistic regression was used to calculate the odds ratio for preeclampsia, followed by log-transformation. A p-value less than 0.05 was considered statistically significant. Since each SNP can represent either a major allele (M) or a minor allele (m), the genotype can be a major allele homozygote (MM), a heterozygote (Mm), or a minor allele homozygote (mm). We therefore performed the comparison of allele frequencies among groups using one of three genetic models: a dominant model (MM versus Mm + mm), a recessive model (MM + Mm versus mm), and a co-dominant model (MM + mm versus Mm). Chi-square tests were used to analyze the ratios in the different groups based on the different genetic models. An R script with reproducible code and detailed comments is provided in the Supplemental Materials (Text S1).

### 4.5. Feature Selection

The original dataset was randomly divided into the training and validation sets under ratio 4:3. The training set was submitted to the feature selection and model building, while the testing set was used to evaluate the models. To account for the imbalance of positive and negative cases in the training set, the R package *imbalance* (version 1.0.2) was used to oversample the smaller population under a ratio of positive to negative cases of 3:5 [64], followed by feature selection using three algorithms: logistic regression, lasso regression, and the Boruta algorithm. The Boruta algorithm was executed using the *Boruta* package (version 7.0.0) under default settings [65]. The Boruta algorithm is a wrapper built around the random forest classification algorithm, in which shadow features are generated from the shuffled values, which are duplicates of the dataset in each column. The features are selected based on the Z-score, ranging from the minimum to the maximum. The lasso regression is based on a regression analysis method, which minimizes the cost function to select those features of use and discard the useless or redundant features, so that it can make its coefficient equal to 0. The logistic regression performs a statistical model that in its basic form uses a logistic function to model a binary dependent variable. Feature importance was calculated as the sum of the decrease in error when split by a feature. Soft thresholds were determined based on the “principle of the mean”, that the importance of a feature should be higher than the mean importance of all features. In this way, the soft threshold was set to 0.5 in lasso regression, 1 in logistic regression, and 2 with the Boruta method. The features that were highlighted by the three methods were then curated manually based on preliminary screening of clinical features using univariable logistic regression analysis, odds ratios, and clinical knowledge. An R script with reproducible code and detailed comments is provided in the Supplemental Materials (Text S1).

### 4.6. Modeling and Evaluation

Machine-learning model building was performed with the *tidymodels* series of packages (https://www.tidymodels.org/) written by the Rstudio team, which includes *tidymodels* (version 0.1.2), *vip* (version 0.3.2), *discrim* (version 0.1.1), *modelr* (version 0.1.8), *yardstick* (version 0.0.7), *workflows* (version 0.2.1), *tune* (version 0.1.2), *rsample* (version 0.0.8), *recipes* (version 0.1.15), and *parsnip* (version 0.1.4). The explanation of the standard protocol is achievable on the website: https://www.tidymodels.org/ (accessed on: 10 August 2021). Meanwhile, an R script with reproducible code and detailed comments is provided in the Supplemental Materials (Text S1) and a package aimed to run this process is under development. Using the features selected in the previous step, we chose eight widely used machine-learning algorithms (elastic net regression, support vector machine, random forest, boost tree, decision tree, k-nearest neighbor, naïve Bayes, and multilayer perceptron) to build and evaluate models. Argument tuning was performed using the 1000-candidate maximum entropy design, an optimal method design of argument combination based on Shannon’s definition of entropy as the amount of information. The oversampled training set was subsequently resampled with five-fold cross-validation, accompanied by two sets of repeats. To evaluate the performance of the models, the receiver operating characteristic (ROC) curves and the area under the receiver operating characteristic curve (AUC) values were used. Specifically, the closer the AUC value is to 1, the better the performance. The quality of each model was also evaluated using metrics of accuracy, sensitivity, specificity, and the adjusted F1-score, which were calculated based on the confusion matrix in Table 4 and Equations (1)–(5). The testing set was retained for final validation.

## 5. Conclusions

By comparing preeclamptic and healthy patients, our study reveals a significant association with a variant of PPARγ (C1431T). By combining data on this variant with information on several clinical features, including the maternal C681G variants, obesity, body mass index, number of pregnancies, primiparity, cigarette use, and education, we built an efficient boost tree model that is able to predict preeclampsia in very early pregnancy. This model could be invaluable in screening high-risk pregnancies in clinical practice and could serve as a decision-making reference for clinicians.

## Figures and Tables

**Table 1:** Maternal clinical features. Values presented as mean and SEM were compared using Student’s t test; values presented as ratios were analyzed using chi-square test. *P*-values < 0.05 indicate a significant difference. SNPs: single nucleotide polymorphisms.

Clinical Features	Controls N = 1613	Preeclampsia N = 35	*P*-Value
Maternal age at delivery (year)	29.6 ± 4.8	28.3 ± 6.1	0.232
Maternal height (cm)	163.0 ± 6.23	165.0 ± 6.35	0.108
Maternal education (level)	6.55 ± 2.47	6.05 ± 2.86	0.358
BMI (before pregnancy, kg/m^2^)	23.2 ± 4.6	25.2 ± 5.8	0.067
Primiparity	44 (703)	60 (21)	0.079
Number of pregnancies	1.35 ± 1.49	1.37 ± 1.77	0.956
Number of deliveries	0.834 ± 0.971	0.657 ± 0.906	0.261
Obesity	9(137)	18(6)	0.109
Cigarette use (no.)	1.48 ± 3.44	1.09 ± 3.37	0.524
**Maternal SNPs (% (N))**			
Pro12Ala	19.7 (333)	26.3 (10)	0.493
C1431T	21.3 (349)	42.9 (15)	0.004
C681G	39.3 (663)	39.5 (15)	1

**Table 2:** Comparison of allele genotypes. Chi-square tests were used to analyze the ratios in the different groups based on the different genetic models.

Maternal SNPs (% (N))	Controls N = 1613			Preeclampsia N = 35			p1 ^a^	p2 ^b^	p3 ^c^
	c/c	c/t	t/t	c/c	c/t	t/t			
C1431T	78.9 (1272)	20.2 (326)	0.9 (15)	57.1 (20)	40 (14)	2.9 (1)	0.004	0.29	0.008
C681G	60.3 (967)	35.2 (565)	4.5 (72)	60 (21)	34.3 (12)	5.7 (2)	1	0.67	1
Pro12Ala	80.4 (1297)	18.7 (301)	0.9 (15)	74.2 (26)	22.9 (8)	2.9 (1)	0.49	0.29	0.68

^a^: p1 for the dominant model. ^b^: p2 for the recessive model. ^c^: p3 for the co-dominant model.

**Table 3:** Prediction of the eight models of machine-learning analysis. The performance of the models was evaluated on the training set first by the accuracy and AUC, followed by the verification on the testing set. The values of the optimal model were bolded. Data were presented mean ± S.D.

	Training Set		Testing Set	
	Accuracy	AUC	Accuracy	AUC
Elastic Net Regression	0.661 ± 0.005	0.695 ± 0.006	0.857	0.784
Random Forest	0.913 ± 0.006	0.969 ± 0.003	0.896	0.723
Support Vector Machine	0.772 ± 0.003	0.847 ± 0.004	0.862	0.545
Decision Tree	0.849 ± 0.007	0.919 ± 0.006	0.874	0.579
K-Nearest Neighbor	0.826 ± 0.006	0.917 ± 0.006	0.801	0.725
Naïve Bayes	0.693 ± 0.005	0.787 ± 0.007	0.930	0.619
**Boost Tree**	**0.971 ± 0.002**	**0.991 ± 0.001**	**0.951**	**0.701**
Multilayer Perceptron	0.899 ± 0.007	0.919 ± 0.006	0.811	0.670

AUC: area under the receiver operating characteristic curve.

**Table 4:** Confusion matrix. Each row of the matrix represents the predicted condition, while each column represents the true condition, which allows more detailed analysis including the accuracy, sensitivity, specificity, precision, and F1-score.

		True Condition	
		Condition Positive	Condition Negative
**Predicted condition**	Predicted condition positive	True positive (TP)	False positive (FP)
	Predicted condition negative	False negative (FN)	True negative (TN)

Accuracy = (TP + TN) / (TP + FN + TN + FP) (1). Sensitivity (Recall) = (TP) / (TP + FN) (2). Specificity = (TN) / (TN + FP) (3). Precision = TP/(TP+FP) (4). F1 = 2× Precision × Recall / (Precision + Recall) (5).

## Data Availability

The datasets used and/or analyzed during the current study are available from the corresponding author on reasonable request.

## Supplementary Materials

The following are available online at www.mdpi.com/xxx/s1.The supplementary materials provide more details about the study and describe additional analyses that were conducted in order to support the results shown in the text. There is information on the procedures that were used to clean up the dataset (Table S1, Figure S1), the evaluation of clinical features (Figure S2), a summary of the odds ratios of clinical features (Table S2), more information on modeling based on machine learning (Tables S3–S6, Figures S3 and S4, Figure S5), and appendix materials regarding parameter tuning (Figure S6).

## References

[1] James M.R., Phyllis A.A., George B., John R.B., Ira M.B., Maurice D., Robert R.G., Joey R.G., Arun J., Donna D.J. (2013). Hypertension in pregnancy. Report of the American College of Obstetricians and Gynecologists’ task force on hypertension in pregnancy. Obstet. Gynecol..

[2] Kuklina E.V., Ayala C., Callaghan W.M. (2009). Hypertensive disorders and severe obstetric morbidity in the United States. Obstet. Gynecol..

[3] Hogan M.C., Foreman K.J., Naghavi M., Ahn S.Y., Wang M., Makela S.M., Lopez A.D., Lozano R., Murray C.J. (2010). Maternal mortality for 181 countries, 1980–2008: A systematic analysis of progress towards Millennium Development Goal 5. Lancet.

[4] Olié V., Moutengou E., Deneux-Tharaux C., Kretz S., Vallée A., Blacher J., Tsatsaris V., Plu-Bureau G. (2020). Prevalence of hypertensive disorders during pregnancy and post-partum in France. Arch. Cardiovasc. Dis. Suppl..

[5] Bahado-Singh R., Poon L.C., Yilmaz A., Syngelaki A., Turkoglu O., Kumar P., Kirma J., Allos M., Accurti V., Li J. (2017). Integrated proteomic and metabolomic prediction of term preeclampsia. Sci. Rep..

[6] Kelly R.S., Croteau-Chonka D.C., Dahlin A., Mirzakhani H., Wu A.C., Wan E.S., McGeachie M.J., Qiu W., Sordillo J.E., Al-Garawi A. (2016). Integration of metabolomic and transcriptomic networks in pregnant women reveals biological pathways and predictive signatures associated with preeclampsia. Metabolomics.

[7] Agrawal S., Cerdeira A.S., Redman C., Vatish M. (2018). Meta-analysis and systematic review to assess the role of soluble FMS-like tyrosine kinase-1 and placenta growth factor ratio in prediction of preeclampsia: The SaPPPhirE study. Hypertension.

[8] Verlohren S., Dröge L.-A. (2020). The diagnostic value of angiogenic and antiangiogenic factors in differential diagnosis of preeclampsia. Am. J. Obstet. Gynecol..

[9] Rana S., Powe C.E., Salahuddin S., Verlohren S., Perschel F.H., Levine R.J., Lim K.-H., Wenger J.B., Thadhani R., Karumanchi S.A. (2012). Angiogenic factors and the risk of adverse outcomes in women with suspected preeclampsia. Circulation.

[10] Romero R., Nien J.K., Espinoza J., Todem D., Fu W., Chung H., Kusanovic J.P., Gotsch F., Erez O., Mazaki-Tovi S. (2008). A longitudinal study of angiogenic (placental growth factor) and anti-angiogenic (soluble endoglin and soluble vascular endothelial growth factor receptor-1) factors in normal pregnancy and patients destined to develop preeclampsia and deliver a small for gestational age neonate. J. Matern. Neonatal Med..

[11] O’Gorman N., Wright D., Syngelaki A., Akolekar R., Wright A., Poon L.C., Nicolaides K.H. (2016). Competing risks model in screening for preeclampsia by maternal factors and biomarkers at 11–13 weeks gestation. Am. J. Obstet. Gynecol..

[12] O’Gorman N.N., Wright D., Poon L.L., Rolnik D.L., Syngelaki A.A., Wright A.A., Akolekar R.R., Cicero S.S., Janga D.D., Jani J. (2017). Accuracy of competing-risks model in screening for pre-eclampsia by maternal factors and biomarkers at 11–13 weeks’ gestation. Ultrasound Obstet. Gynecol..

[13] McGinnis R., Steinthorsdottir V., Williams N.O., Thorleifsson G., Shooter S., Hjartardottir S., Bumpstead S., Stefansdottir L., Hildyard L., Sigurdsson J.K. (2017). Variants in the fetal genome near FLT1 are associated with risk of preeclampsia. Nat. Genet..

[14] Gray K.J., Kovacheva V.P., Mirzakhani H., Bjonnes A.C., Almoguera B., DeWan A.T., Triche E.W., Saftlas A.F., Hoh J., Bodian D.L. (2018). Gene-centric analysis of preeclampsia identifies maternal association at PLEKHG1. Hypertension.

[15] Meirhaeghe A., Fajas L., Helbecque N., Cottel D., Lebel P., Dallongeville J., Deeb S., Auwerx J., Amouyel P. (1998). A genetic polymorphism of the peroxisome proliferator-activated receptor γ gene influences plasma leptin levels in obese humans. Hum. Mol. Genet..

[16] Doney A., Fischer B., Frew D., Cumming A., Flavell D.M., World M., Montgomery H.E., Boyle D., Morris A., Palmer C.N. (2002). Haplotype analysis of the PPARγ Pro12Ala and C1431T variants reveals opposing associations with body weight. BMC Genet..

[17] Valve R., Sivenius K., Miettinen R., Pihlajamaki J., Rissanen A., Deeb S.S., Auwerx J., Uusitupa M., Laakso M. (1999). Two polymorphisms in the peroxisome proliferator-activated receptor-γ gene are associated with severe overweight among obese women. J. Clin. Endocrinol. Metab..

[18] Cai G., Zhang X., Weng W., Shi G., Xue S., Zhang B. (2017). Associations between PPARG polymorphisms and the risk of essential hypertension. PLoS ONE.

[19] Chao T.H., Li Y.H., Chen J.H., Wu H.L., Shi G.Y., Liu P.Y., Tsai W.C., Guo H.R. (2004). The 161TT genotype in the exon 6 of the peroxisome-proliferator-activated receptor γ gene is associated with premature acute myocardial infarction and increased lipid peroxidation in habitual heavy smokers. Clin. Sci. Lond..

[20] Wang X.L., Oosterhof J., Duarte N. (1999). Peroxisome proliferator-activated receptor γ C161 → T polymorphism and coronary artery disease. Cardiovasc. Res..

[21] Lin J., Chen Y., Tang W.-F., Liu C., Zhang S., Guo Z.-Q., Chen G., Zheng X.-W. (2019). PPARG rs3856806 C> T polymorphism increased the risk of colorectal cancer: A case-control study in Eastern Chinese Han population. Front. Oncol..

[22] Heude B., Pelloux V., Forhan A., Bedel J.F., Lacorte J.M., Clement K., Charles M.A., EDEN Mother-Child Cohort Study Group (2011). Association of the Pro12Ala and C1431T variants of PPARγ and their haplotypes with susceptibility to gestational diabetes. J. Clin. Endocrinol. Metab..

[23] Gannoun M., Raguema N., Zitouni H., Mehdi M., Seda O., Mahjoub T., Lavoie J. (2021). MMP-2 and MMP-9 polymorphisms and preeclampsia risk in Tunisian Arabs: A case-control study. J. Clin. Med..

[24] Pinto-Souza C.C., Coeli-Lacchini F., Luizon M.R., Cavalli R.C., Lacchini R., Sandrim V.C. (2021). Effects of arginase genetic polymorphisms on nitric oxide formation in healthy pregnancy and in preeclampsia. Nitric Oxide.

[25] Gray K.J., Kovacheva V.P., Mirzakhani H., Bjonnes A.C., Almoguera B., Wilson M.L., Ingles S.A., Lockwood C.J., Hakonarson H., McElrath T.F. (2021). Risk of pre-eclampsia in patients with a maternal genetic predisposition to common medical conditions: A case–control study. BJOG Int. J. Obstet. Gynaecol..

[26] Azhar S. (2010). Peroxisome proliferator-activated receptors, metabolic syndrome and cardiovascular disease. Future Cardiol..

[27] Peng L., Yang H., Ye Y., Ma Z., Kuhn C., Rahmeh M., Mahner S., Makrigiannakis A., Jeschke U., von Schönfeldt V. (2021). Role of peroxisome proliferator-activated receptors (PPARs) in trophoblast functions. Int. J. Mol. Sci..

[28] Duan S.Z., Ivashchenko C.Y., Whitesall S.E., D’Alecy L.G., Duquaine D.C., Brosius F.C., Gonzalez F.J., Vinson C., Pierre M.A., Milstone D.S. (2007). Hypotension, lipodystrophy, and insulin resistance in generalized PPARγ-deficient mice rescued from embryonic lethality. J. Clin. Investig..

[29] Barak Y., Nelson M.C., Ong E.S., Jones Y.Z., Ruiz-Lozano P., Chien K.R., Koder A., Evans R.M. (1999). PPAR γ is required for placental, cardiac, and adipose tissue development. Mol. Cell.

[30] McCarthy F.P., Drewlo S., English F.A., Kingdom J., Johns E.J., Kenny L.C., Walsh S.K. (2011). Evidence implicating peroxisome proliferator-activated receptor-γ in the pathogenesis of preeclampsia. Hypertension.

[31] Almeida S.M., Furtado J.M., Mascarenhas P., Ferraz M.E., Ferreira J.C., Monteiro M., Vilanova M., Ferraz F.P. (2018). Association between LEPR, FTO, MC4R, and PPARG-2 polymorphisms with obesity traits and metabolic phenotypes in school-aged children. Endocrine.

[32] Jiang J., Xie Z., Guo J., Wang Y., Liu C., Zhang S., Tang W., Chen Y. (2017). Association of PPARG rs 1801282 C> G polymorphism with risk of colorectal cancer: From a case-control study to a meta-analysis. Oncotarget.

[33] Rocha R.M., Barra G., Rosa É.C., Garcia É.C., Amato A.A., Azevedo M.F. (2015). Prevalence of the rs1801282 single nucleotide polymorphism of the PPARG gene in patients with metabolic syndrome. Arch. Endocrinol. Metab..

[34] Ho J.S., Germer S., Tam C.H., So W.-Y., Martin M., Ma R.C., Chan J., Ng M.C. (2012). Association of the PPARG Pro12Ala polymorphism with type 2 diabetes and incident coronary heart disease in a Hong Kong Chinese population. Diabetes Res. Clin. Pr..

[35] Deeb S.S., Fajas L., Nemoto M., Pihlajamaki J., Mykkanen L., Kuusisto J., Laakso M., Fujimoto W., Auwerx J. (1998). A Pro12Ala substitution in PPARγ2 associated with decreased receptor activity, lower body mass index and improved insulin sensitivity. Nat. Genet..

[36] Altshuler D., Hirschhorn J.N., Klannemark M., Lindgren C.M., Vohl M.C., Nemesh J., Lane C.R., Schaffner S.F., Bolk S., Brewer C. (2000). The common PPARγ Pro12Ala polymorphism is associated with decreased risk of type 2 diabetes. Nat. Genet..

[37] Beamer B.A., Yen C.-J., Andersen R.E., Muller D., Elahi D., Cheskin L.J., Andres R., Roth J., Shuldiner A. (1998). Association of the Pro12Ala variant in the peroxisome proliferator-activated receptor-γ2 gene with obesity in two Caucasian populations. Diabetes.

[38] Cecil J.E., Fischer B., Doney A.S.F., Hetherington M., Watt P., Wrieden W., Bolton-Smith C., Palmer C.N.A. (2005). The Pro12Ala and C–681G variants of the PPARG locus are associated with opposing growth phenotypes in young schoolchildren. Diabetologia.

[39] Heude B., Forhan A., Slama R., Douhaud L., Bedel S., Saurel-Cubizolles M.-J., Hankard R., Thiebaugeorges O., de Agostini M., Annesi-Maesano I. (2016). Cohort profile: The EDEN mother-child cohort on the prenatal and early postnatal determinants of child health and development. Int. J. Epidemiol..

[40] Permadi W., Mantilidewi K.I., Khairani A.F., Lantika U.A., Ronosulistyo A.R., Bayuaji H. (2020). Differences in expression of peroxisome proliferator-activated receptor-γ in early-onset preeclampsia and late-onset preeclampsia. BMC Res. Notes.

[41] Kadam L., Kohan-Ghadr H.R., Drewlo S. (2015). The balancing act—PPAR-γ’s roles at the maternal-fetal interface. Syst. Biol. Reprod. Med..

[42] McCarthy F.P., Drewlo S., Kingdom J., Johns E.J., Walsh S.K., Kenny L.C. (2011). Peroxisome proliferator-activated receptor-γ as a potential therapeutic target in the treatment of preeclampsia. Hypertension.

[43] Ganss R. (2017). Maternal metabolism and vascular adaptation in pregnancy: The PPAR link. Trends Endocrinol. Metab..

[44] Laasanen J., Heinonen S., Hiltunen M., Mannermaa A., Laakso M. (2002). Polymorphism in the peroxisome proliferator-activated receptor-γ gene in women with preeclampsia. Early Hum. Dev..

[45] Hoffman M.K., Ma N., Roberts A. (2021). A machine learning algorithm for predicting maternal readmission for hypertensive disorders of pregnancy. Am. J. Obstet. Gynecol. MFM.

[46] Sufriyana H., Wu Y.-W., Su E.C.-Y. (2020). Artificial intelligence-assisted prediction of preeclampsia: Development and external validation of a nationwide health insurance dataset of the BPJS Kesehatan in Indonesia. EBioMedicine.

[47] Bodnar L.M., Cartus A.R., Kirkpatrick S.I., Himes K.P., Kennedy E.H., Simhan H.N., Grobman W.A., Duffy J.Y., Silver R.M., Parry S. (2020). Machine learning as a strategy to account for dietary synergy: An illustration based on dietary intake and adverse pregnancy outcomes. Am. J. Clin. Nutr..

[48] Jhee J.H., Lee S., Park Y., Lee S.E., Kim Y.A., Kang S.-W., Kwon J.-Y., Park J.T. (2019). Prediction model development of late-onset preeclampsia using machine learning-based methods. PLoS ONE.

[49] (2019). ACOG practice bulletin no. 202 summary: Gestational hypertension and preeclampsia. Obstet. Gynecol..

[50] (2015). Committee opinion no. 638: First-trimester risk assessment for early-onset preeclampsia. Obstet. Gynecol..

[51] Brasier A., Victor S., Ju H., Busse W.W., Curran-Everett U., Bleecker E., Castro M., Chung K.F., Gaston B., Israel E. (2010). Predicting intermediate phenotypes in asthma using bronchoalveolar lavage-derived cytokines. Clin. Transl. Sci..

[52] Dreiseitl S., Ohno-Machado L. (2002). Logistic regression and artificial neural network classification models: A methodology review. J. Biomed. Inform..

[53] Kuhle S., Maguire B., Zhang H., Hamilton D., Allen A.C., Joseph K.S., Allen V.M. (2018). Comparison of logistic regression with machine learning methods for the prediction of fetal growth abnormalities: A retrospective cohort study. BMC Pregnancy Childbirth.

[54] Luque A., Carrasco A., Martín A., de las Heras A. (2019). The impact of class imbalance in classification performance metrics based on the binary confusion matrix. Pattern Recognit..

[55] Banerjee P., Dehnbostel F.O., Preissner R. (2018). Prediction is a balancing act: Importance of sampling methods to balance sensitivity and specificity of predictive models based on imbalanced chemical data sets. Front. Chem..

[56] Gorman N.O., Wright D., Poon L.C., Rolnik D.L., Syngelaki A., de Alvarado M., Carbone I.F., Dutemeyer V., Fiolna M., Frick A. (2017). Multicenter screening for pre-eclampsia by maternal factors and biomarkers at 11–13 weeks’ gestation: Comparison with NICE guidelines and ACOG recommendations. Ultrasound Obstet. Gynecol..

[57] Rana S., Salahuddin S., Mueller A., Berg A.H., Thadhani R.I., Karumanchi S.A. (2018). Angiogenic biomarkers in triage and risk for preeclampsia with severe features. Pregnancy Hypertens..

[58] Odibo A.O., Goetzinger K.R., Odibo L., Cahill A.G., Macones G.A., Nelson D.M., Dietzen D.J. (2011). First-trimester prediction of preeclampsia using metabolomic biomarkers: A discovery phase study. Prenat. Diagn..

[59] Bahado-Singh R.O., Syngelaki A., Akolekar R., Mandal R., Bjondahl T.C., Han B., Dong E., Bauer S., Alpay-Savasan Z., Graham S. (2015). Validation of metabolomic models for prediction of early-onset preeclampsia. Am. J. Obstet. Gynecol..

[60] Ding S., Liu L., Zhuge Q.-C., Yu Z., Zhang X., Xie J., Hong W., Wang S., Yang Y., Chen B. (2012). The meta-analysis of the association of PPARG P12A, C161T polymorphism and coronary heart disease. Wien. Klin. Wochenschr..

[61] Van Buuren S., Groothuis-Oudshoorn K. (2011). Mice: Multivariate imputation by chained equations in R. J. Stat. Softw..

[62] Stekhoven D.J., Buhlmann P. (2012). MissForest—Non-parametric missing value imputation for mixed-type data. Bioinformatics.

[63] Lê S., Josse J., Husson F. (2008). FactoMineR: An R package for multivariate analysis. J. Stat. Softw..

[64] Cordón I., García S., Fernández A., Herrera F. (2018). Imbalance: Oversampling algorithms for imbalanced classification in R. Knowl.-Based Syst..

[65] Kursa M., Rudnicki W. (2010). Feature selection with the Boruta package. J. Stat. Softw..

