# Supplementary material for: C1431T Variant of PPARγ Is Associated with Preeclampsia in Pregnant Women
Source: Life (Basel). 2021 Oct 7;11(10):1052. doi: 10.3390/life11101052 (PMC8540421; doi:10.3390/life11101052)
Supplement: Supplementary file 1 [file life-11-01052-s001.zip › life-1360167-supplementary.pdf]

life

Supplementary

C1431T Variant of PPARγ Is Associated with Preeclampsia in Pregnant Women

Fulin Liu [1], Christine Rouault [2], Karine Clément [2,3], Wencan Zhu [4], Séverine A. Degrelle [1,5], Marie-Aline Charles [6,7], Barbara Heude [6,*] and Thierry Fournier [1,*]

1 Pathophysiology & Pharmacotoxicology of the Human Placenta, Pre & Postnatal Microbiota, 3PHM, INSERM, Université de Paris, F-75006, Paris, France; fulin.liu@etu.u-paris.fr (F.L.); severine.degrelle@inserm.fr (S.A.D.)
2 Nutrition et Obesités: Approches Systémiques Research Unit, INSERM, Sorbonne Université, F-75013 Paris, France; christine.rouault@nutriomique.org (C.R.); karine.clement@psl.aphp.fr (K.C.)
3 Nutrition Department, Pitié-Salpêtrière hospital, Assistance Publique hôpitaux de Paris, F-75013 Paris, France
4 UMR MIA-Paris, INRAE, AgroParisTech, Université Paris-Saclay, 75005 Paris, France; wencan.zhu@agroparistech.fr
5 Inovarion, F-75005 Paris, France
6 Centre for Research in Epidemiology and Statistics (CRESS), INSERM, INRAE, Université de Paris, F-75004 Paris, France; marie-aline.charles@inserm.fr (M.-A.C.)
7 Ined, Unité Mixte Inserm-Ined-EFS ELFE, F-75020 Paris, France
* Correspondence: barbara.heude@inserm.fr (B.H.); thierry.fournier@inserm.fr (T.F.)

Citation: Liu, F.; Rouault, C.; Clément, K.; Zhu, W.; Degrelle, S.A.; Charles, M.-A.; Heude, B.; Fournier, T. C1431T Variant of PPARγ Is Associated with Preeclampsia in Pregnant Women. *Life* **2021**, *11*, 1052. https://doi.org/10.3390/life11101052

Academic Editors: Ilona Hromadnikova and Katerina Kotlabova

Received: 13 August 2021
Accepted: 2 October 2021
Published: 7 October 2021

Publisher's Note: MDPI stays neutral with regard to jurisdictional claims in published maps and institutional affiliations.

Copyright: © 2021 by the authors. Licensee MDPI, Basel, Switzerland. This article is an open access article distributed under the terms and conditions of the Creative Commons Attribution (CC BY) license (http://creativecommons.org/licenses/by/4.0/).

The supplementary materials provide more details about the analysis process and describe additional analyses that were conducted in order to support the results shown in the text. There is information on the procedures that were used to clean up the dataset (Table S1, Figure S1), the evaluation of clinical characteristics (Figure S2), a summary of the odds ratios of clinical characteristics (Table S2), more information on modeling based on machine learning (Table S3–6, Figure S3&S4, Figure S5), and appendix materials regarding parameter tuning (Figure S6). The F−score was also calculated to overcome the possible inadequacy of accuracy as a metric in the final fetal feature−free models (Table S6).

1. Dataset Tidying

Different with the data frame containing only the maternal characteristics in the manuscript, we included the clinical characteristics of both the mother and the fetus. Before the analyses, we performed the imputation to the missing values instead of simple deletion. The missing data plot shows the distribution of non−available (NA) data (Figure S1). The completed observations are 1057 while the rest 591 have different degree of deficiency in the features. The total NAs count on 9.14% percent (NAs to total cells) of the full data frame, which is perfectly less than the threshold of 10 percent for data imputation. The comparison of the data before and after imputation was showed in Table S1, with respect to the feature types. No difference was shown between them.

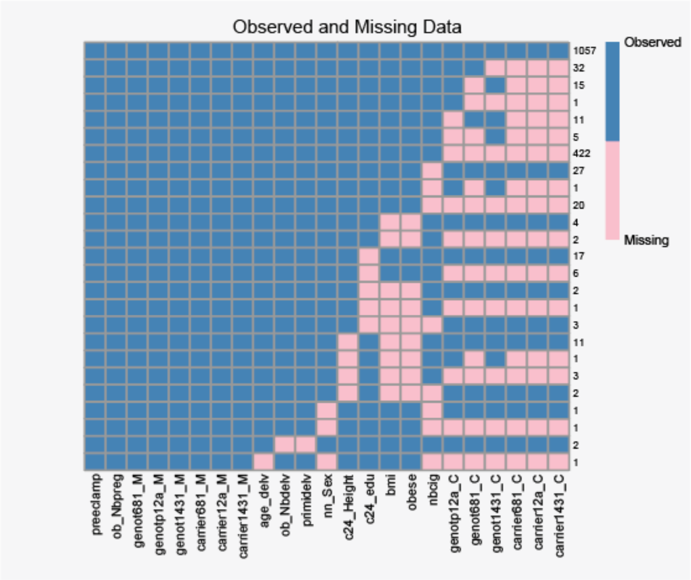

Figure S1. Overview of missing data in original data. Columns represent clinical characteristics. Rows indicate the missing characteristics and the corresponding number of individuals. Pink grids represent missing values while blue grids represent observed values.

Table S1. S. Comparison of imputation for factor type features before and after. *continued.*

Features	Number of Missing Values	Imputation	
		Before<br>Count of Factors	After<br>Count of Factors
preeclamp	0	0: 1613, 1: 35	0: 1613, 1: 35
nn_Sex	3	1: 878, 2: 767	1: 879, 2: 769
primidelv	2	0: 922, 1: 724	0: 924, 1: 724
obese	29	0: 1476, 1: 143	0: 1505, 1: 143
genot681_C	479	11: 698, 12: 419, 22: 52	11: 1175, 12: 420, 22: 53
genot681_M	0	11: 997, 12: 577, 22: 74	11: 997, 12: 577, 22: 74
genotp12a_C	472	11: 946, 12: 219, 22: 11	11: 1418, 12: 219, 22: 11
genotp12a_M	0	11: 1323, 12: 309, 22: 16	11: 1323, 12: 309, 22: 16
genot1431_C	489	11: 880, 12: 264, 22: 15	11: 1369, 12: 264, 22: 15
genot1431_M	0	11: 1292, 12: 340, 22: 16	11: 1292, 12: 340, 22: 16
carrier681_M	0	0: 997, 1: 651	0: 997, 1: 651
carrier12a_M	0	0: 1323, 1: 325	0: 1323, 1: 325
carrier1431_M	0	0: 1292, 1: 356	0: 1292, 1: 356
carrier681_C	522	0: 666, 1: 460	0: 1175, 1: 473
carrier12a_C	522	0: 906, 1: 220	0: 1418, 1: 230
carrier1431_C	522	0: 854, 1: 272	0: 1369, 1: 279

Table S2. Comparison of imputation for numeric type features before and after.

	Number of Missing Values	Imputation			
		Before		After	
		Mean	sd	Mean	sd
c24_Height	17	163.46	6.23	163.46	6.20
c24_edu	29	6.54	2.48	6.53	2.47
ob_Nbpreg	0	1.35	1.49	1.35	1.49
ob_Nbdelv	2	0.83	0.97	0.83	0.97

bmi	29	23.27	4.63	23.26	4.60
age_delv	1	29.55	4.86	29.55	4.86
nbcig	56	1.47	3.44	1.48	3.39

2. Evaluation of Clinical Characteristics

With the imputed data, we evaluated the features (clinical characteristics) using principal component analysis (PCA) and logistic regression. Firstly, the correlation was explored for all the features, which shows the strong correlation of the number of deliveries and number of pregnancies and medium correlation among the polymorphisms (Figure S2A). Secondly, the weightiness of features was analyzed by the PCA with the pre–ranked features counting about 30% in first and second dimensions, which shows the considerable importance of genetic features (Figure S2B). However, the low total proportion indicates the difficulties in representing individuals by only several features, which was confirmed by the overlapped distribution of individuals between groups through PCA and t–SNE analyses (Figure S2C&S2D). Additionally, the distribution of balanced individuals by t–SNE in the first dimensions was shown to compare with the original, which indicated the general similar distribution of data points. Table S2 presents the log odds ratio of maternal and fetal clinical characteristics of the control and preeclampsia groups. The value of the 95% confidence interval odds ratio value for the characteristics were also calculated.

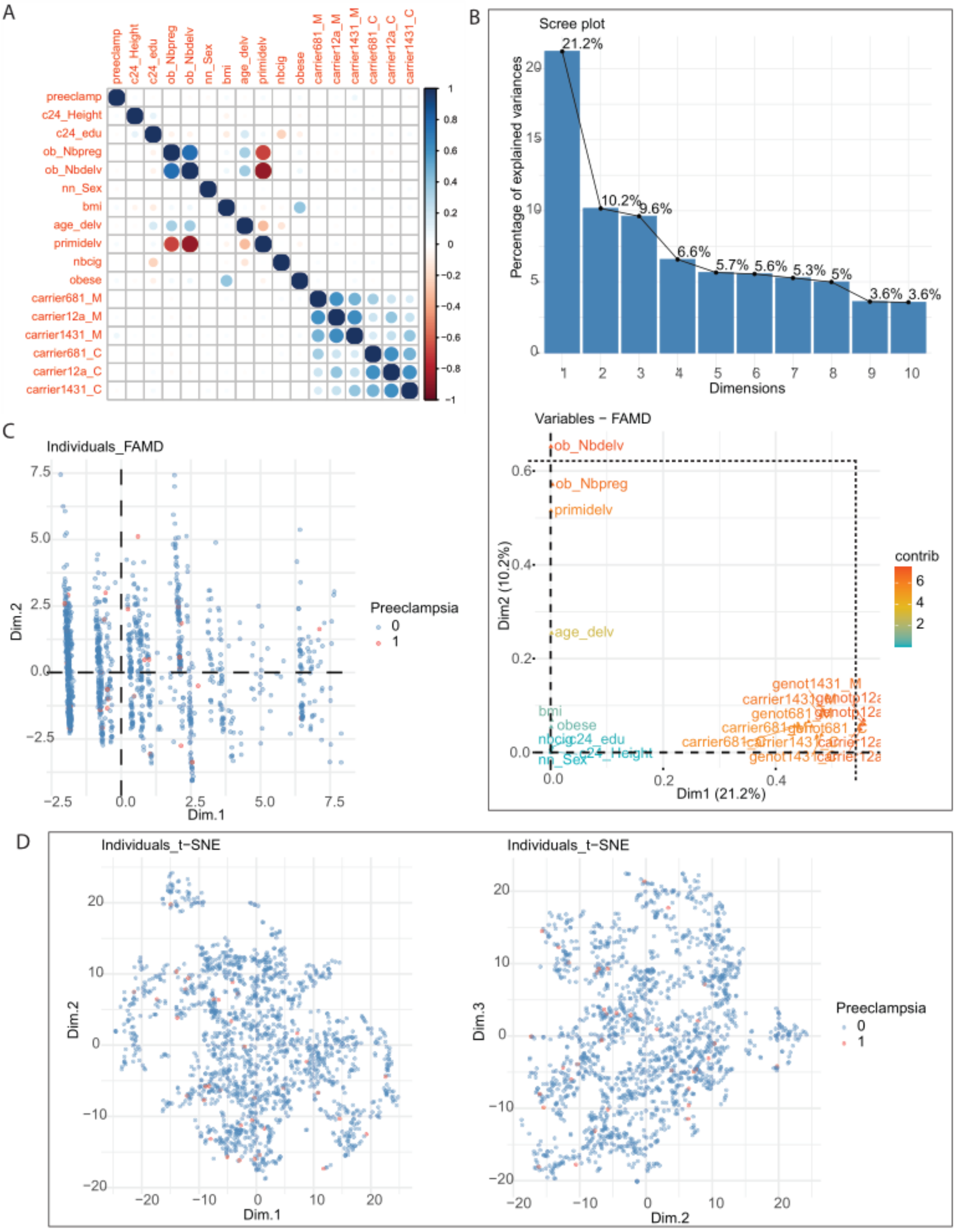
A
B
Scree plot
Percentage of explained variances
Dimensions
21.2%
10.2%
9.6%
6.6%
5.7%
5.6%
5.3%
5%
3.6%
3.6%
C
Individuals_FAMD
Dim.1
Dim.2
Preeclampsia
Variables - FAMD
Dim1 (21.2%)
Dim2 (10.2%)
contrib
ob_Nbdelv
ob_Nbpreg
primidelv
age_delv
bmi
obese
nbcig
c24_edu
nn_Sex
c24_Height
preeclamp
carrier681_M
carrier12a_M
carrier1431_M
carrier681_C
carrier12a_C
carrier1431_C
D
Individuals_t-SNE
Individuals_t-SNE
Dim.3

E

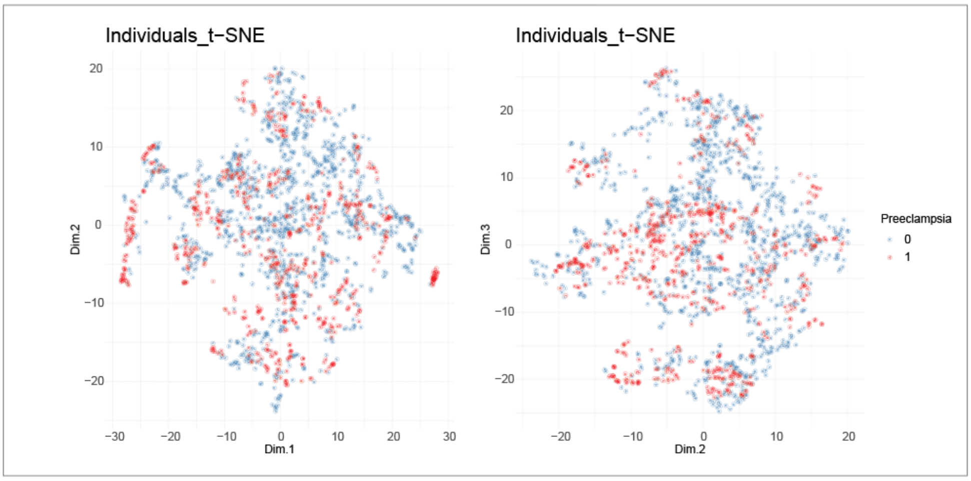

Figure S2. Evaluation of clinical characteristics. (A) The matrix of correlation coefficient for all the characteristics. The factorial characteristics were transformed to numerical types, accompanied by adding 0.1 to all the data in order to facilitate the calculation without 0. (B) A principal component analysis with factor analysis of mixed data (FAMD) for all the characteristics with detailed component proportions shown in the right. The first and second dimensions were chosen to draw the graph with colors presenting the contribution. (C) Distribution of individuals by FAMD. (D) Distribution of individuals by t–SNE in the dimensions before balancing. (E) Distribution of individuals by t–SNE in the dimensions after balancing.

Table S3. Summary of odds ratio.

	Log.OR	Std.Error	95% CI of Log.OR	95% CI of OR	P value
(Intercept)	−7.45	1.33	(−10.08, −4.85)	(0.00004, 0.0078)	2.33E−08
c24_Height	0.033	0.0075	(0.018, 0.047)	(1.02, 1.05)	1.44E−05
c24_edu	−0.079	0.020	(−0.12, −0.04)	(0.89, 0.96)	6.18E−05
ob_Nbpreg	0.33	0.054	(0.22, 0.43)	(1.25, 1.54)	1.00E−09
ob_Nbdelv	−0.45	0.12	(−0.70, −0.22)	(0.50, 0.80)	0.000191
nn_Sex	−0.46	0.094	(−0.65, −0.28)	(0.52, 0.76)	9.84E−07
bmi	0.085	0.014	(0.058, 0.11)	(1.06, 1.12)	1.66E−09
age_delv	−0.014	0.011	(−0.034, 0.0072)	(0.97, 1.01)	0.201581
primidelv	0.77	0.17	(0.45, 1.10)	(1.56, 3.00)	3.03E−06
nbcig	−0.071	0.016	(−0.10, −0.039)	(0.90, 0.96)	1.36E−05
obese	0.085	0.21	(−0.33, 0.50)	(0.72, 1.65)	0.686189
carrier681_M	−0.76	0.14	(−1.05, −0.49)	(0.35, 0.62)	9.41E−08
carrier12a_M	−0.29	0.18	(−0.65, 0.069)	(0.52, 1.07)	0.114834
carrier1431_M	1.87	0.15	(1.59, 2.17)	(4.90, 8.75)	1.07E−36
carrier681_C	0.47	0.15	(0.18, 0.76)	(1.20, 2.15)	0.001383
carrier12a_C	0.57	0.21	(0.16, 0.98)	(1.18, 2.65)	0.006159
carrier1431_C	−1.69	0.19	(−2.07, −1.31)	(0.13, 0.27)	2.15E−18

3. Modeling Based on Machine Learning

3.1. Overview of Maternal and Fetal Clinical Characteristics in Training and Testing Sets

With the selected features, we divided the dataset into two parts, training set and testing set. The clinical characteristics of the original dataset and the split dataset were shown in Table S3, with respect to the feature types. The proportion of the factors in each factor features is equal in total, training and testing set, as same as the mean and standard deviation of the numeric features. In order to obtain the optimal performance of each

model, the arguments of each model were tuned and the optimal combination was selected according to the highest AUC value. The combination of arguments and their performances were shown in Figure S6 in the appendix.

Table S3. Clinical characteristics for different data set related to factor features. *continued.*

Factor Features	Number of Factors	Total	Train	Test
		Count of Factors	Count of Factors	Count of Factors
nn_Sex	2	1: 879, 2: 769	1: 660, 2: 576	1: 220, 2: 192
primidelv	2	0: 924, 1: 724	0: 713, 1: 523	0: 211, 1: 201
preeclamp	2	0: 1613, 1: 35	0: 1211, 1: 25	0: 402, 1: 10
genot681_C	3	11: 1175, 12: 420, 22: 53	11: 875, 12: 324, 22: 37	11: 300, 12: 97, 22: 15
genot681_M	3	11: 997, 12: 577, 22: 74	11: 753, 12: 420, 22: 63	11: 244, 12: 157, 22: 11
genot1431_C	3	11: 1369, 12: 264, 22: 15	11: 1027, 12: 196, 22: 13	11: 342, 12: 68, 22: 2
genot1431_M	3	11: 1292, 12: 340, 22: 16	11: 973, 12: 250, 22: 13	11: 319, 12: 90, 22: 3
obese	2	0: 1505, 1: 143	0: 1120, 1: 116	0: 384, 1: 28

Table S3. Clinical characteristics for different data set related to numeric features.

Numeric Features	Total		Train		Test	
	Mean	sd	Mean	sd	Mean	sd
c24_edu	6.53	2.47	6.50	2.48	6.59	2.43
ob_Nbpreg	1.35	1.49	1.42	1.54	1.15	1.32
bmi	23.26	4.60	23.40	4.67	22.93	4.43
nbcig	1.48	3.39	1.57	3.49	1.24	3.07

3.2. Modeling with or without Oversampling in Dataset

To exclude the possibility of overfit in our models, different approaches were used to preprocess our dataset. Firstly, the original dataset was split into training set and testing set directly as shown in Table S3, followed by the model building with training set solely and validation in testing set. The result showed the most outstanding model remained the boost tree with the accuracy and AUC value being 0.99 and 0.92 in training set, and 0.98 and 0.77 in testing set (Table S4 & Figure S3). However, considering that the imbalance of positive cases (37) and negative cases (1611) in our dataset would affect the accuracy of the models, we secondly balanced the training set by oversampling the positive cases, followed by the validation in testing set, as shown in the manuscript (Table 3 & Figure 4). Lastly, we balanced the original dataset before splitting into training set and testing set. The optimal model remained the boost tree with the accuracy and AUC value being 0.957 and 0.990 in training set, and 0.975 and 0.996 in testing set (Table S5 & Figure S4). Herein, we speculated the existence of overfit in the model owing to the internal relationship between the training set and the testing set that resulted from the data simulation.

Table S4. Prediction of the 8 Models by ML Analysis (without balancing).

	Train		Test	
	**Accuracy**	**AUC**	**Accuracy**	**AUC**
Elastic Net Regression	0.98	0.70	0.98	0.76
Random Forest	0.99	0.84	0.98	0.73
Support Vector Machine	0.99	0.89	0.98	0.66
Decision Tree	0.98	0.82	0.97	0.54
K−Nearest Neighbor	0.97	0.66	0.95	0.51
Naïve Bayes	0.99	0.74	0.97	0.63
**Boost Tree**	**0.99**	**0.92**	**0.98**	**0.77**
Multilayer Perceptron	0.98	0.82	0.95	0.55

AUC, area under the receiver operating characteristic curve.

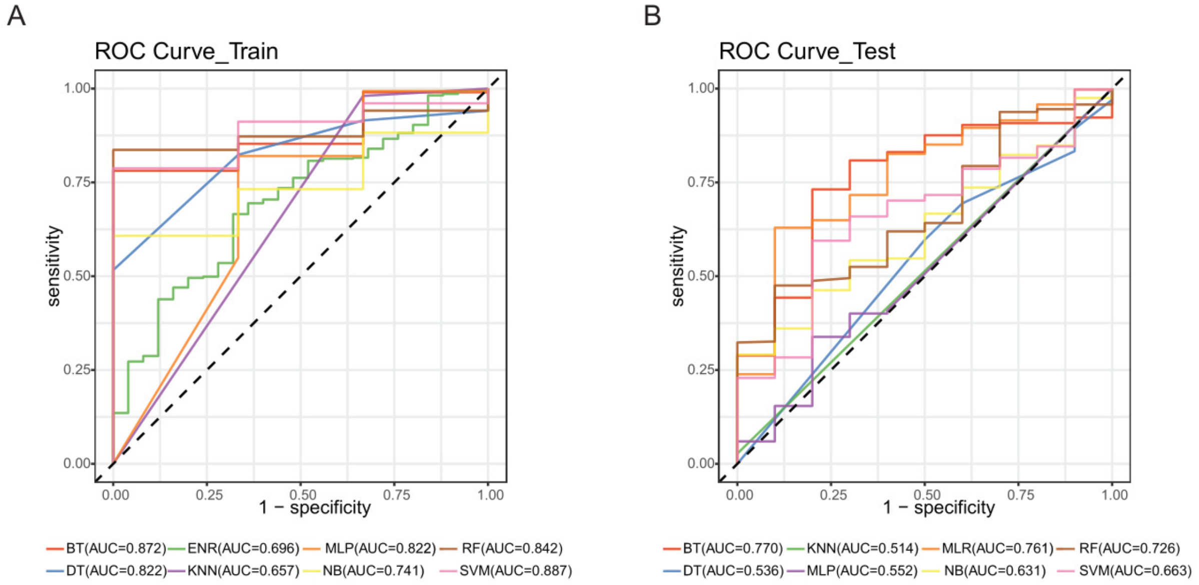

Figure S3. ROC curve of different algorithms. (A) ROC curves with training set. (B) ROC curves with testing set. The values were shown in the legends. AUC: area under the receiver operating characteristic curve; BT: boost tree; DT: decision tree; ENR: elastic net regression; KNN: k−nearest neighbor; MLP: multilayer perceptron; NB: naïve bayes; RF: random forest; SVM: support vector machine; ROC: receiver operating characteristic.

Table S5. Prediction of the 8 Models by ML Analysis (with total balancing before split).

	Train		Test	
	**Accuracy**	**AUC**	**Accuracy**	**AUC**
Elastic Net Regression	0.721	0.761	0.705	0.735
Random Forest	0.911	0.966	0.913	0.973
Support Vector Machine	0.783	0.864	0.743	0.807
Decision Tree	0.863	0.926	0.819	0.892
K−Nearest Neighbor	0.830	0.901	0.772	0.876
Naïve Bayes	0.687	0.770	0.702	0.787
**Boost Tree**	**0.957**	**0.990**	**0.975**	**0.996**
Multilayer Perceptron	0.863	0.926	0.735	0.800

AUC, area under the receiver operating characteristic curve.

A

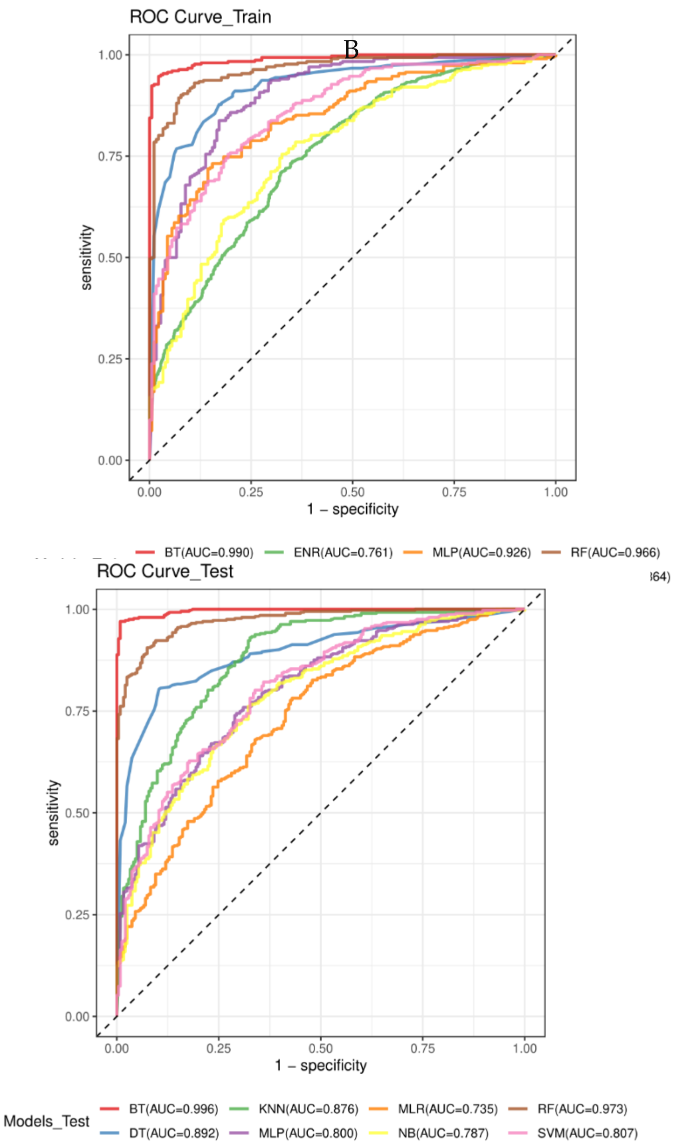

Figure S4. ROC curve of different algorithms. (A) ROC curves with training set. (B) ROC curves with testing set. The values were shown in the legends. AUC: area under the receiver operating characteristic curve; BT: boost tree; DT: decision tree; ENR: elastic net regression; KNN: k−nearest neighbor; MLP: multilayer perceptron; NB: naïve bayes; RF: random forest; SVM: support vector machine; ROC: receiver operating characteristic.

3.3. Modeling with or without Fetal Characteristics in Dataset

Furthermore, considering the difficult clinical situation in collecting fetal genotype than that of the maternal, we thus excluded the fetal features (fetal genotypes and sex) in the predictive model. We manually excluded the fetal features (fetal genotypes and sex) for the following predictive models for two reasons. First, to facilitate the prediction since the fetal genotype is not easily accessible as that of the maternal one; Second, to bring forward the time of preeclampsia evaluation without the limitation of fetal features, we compared the models with and without fetal features. The results showed that fetal−feature−free models generally had better accuracy and AUC values than models with fetal features, either in balanced dataset or non−balanced dataset (Figure S5). Therefore, the final included features for the model building included the maternal carrying of C681G and C1341T, obesity, BMI, number of pregnancies, primary delivery, number of cigarettes,

and education. The F−score was also calculated to overcome the possible inadequacy of accuracy as a metric in the final fetal feature−free models (Table S6).

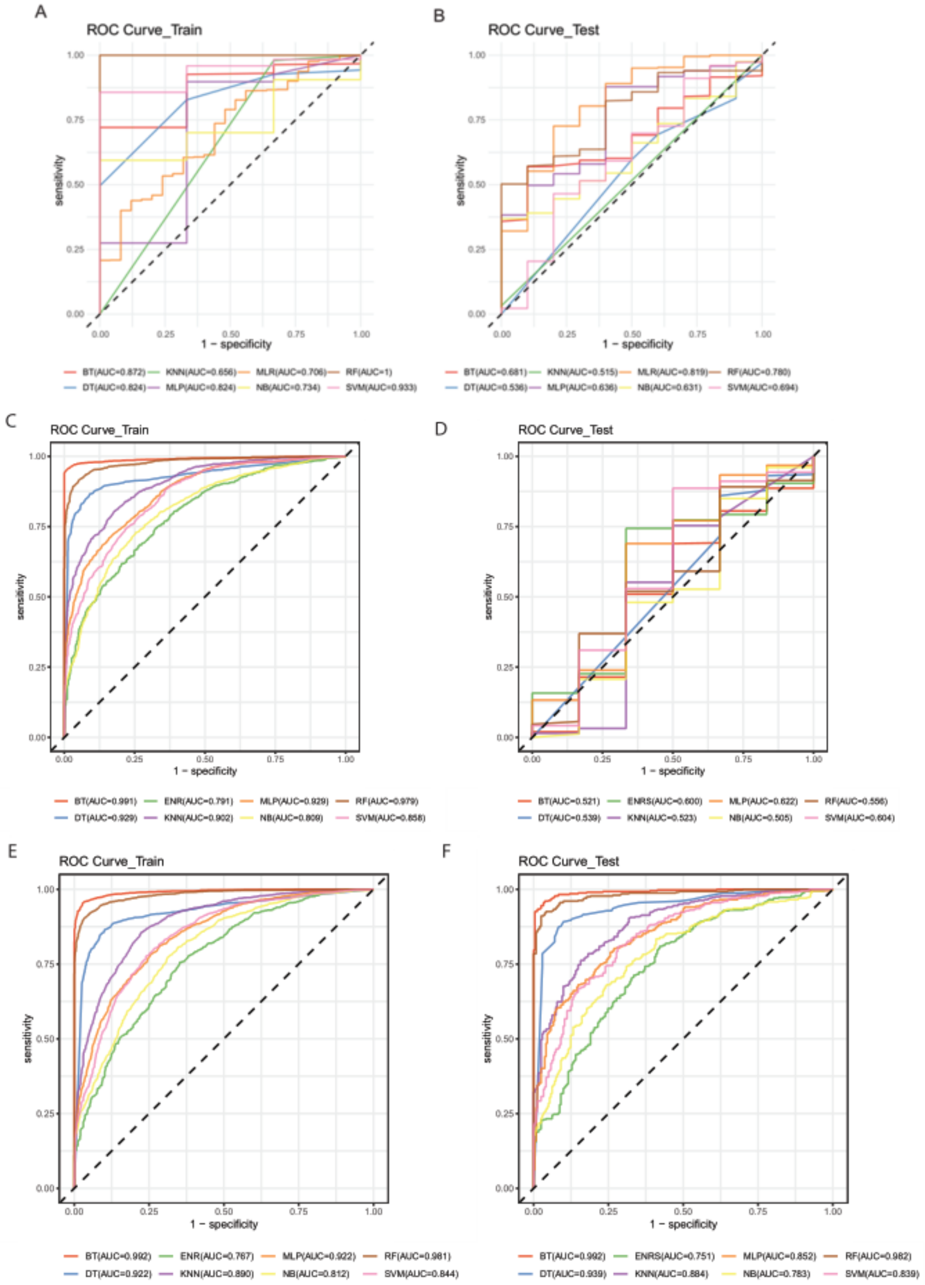

Figure S5. ROC curve of different algorithms before and after balancing in fetal−feature−including models. (A&B) ROC curves with training set and testing set without balancing. (C&D) ROC curves with training set balanced only. (E&F) ROC curves with training set and testing set both balanced. The values were shown in the legends. AUC: area under the receiver operating characteristic curve; BT: boost tree; DT: decision tree; ENR: elastic net regression; KNN: k−nearest neighbor; MLP: multilayer perceptron; NB: naïve bayes; RF: random forest; SVM: support vector machine; ROC: receiver operating characteristic.

Table S6. F−scores of fetal feature−free models.

	Train	Test
Elastic Net Regression	0.768	0.922
Random Forest	0.997	0.945
Support Vector Machine	0.846	0.925
Decision Tree	0.945	0.933
K−Nearest Neighbor	0.998	0.888
Naïve Bayes	0.808	0.963
**Boost Tree**	0.990	0.975
Multilayer Perceptron	0.853	0.894

3.4. Modeling with Undersampling Method

Besides, even though appropriate algorithm has been used to deal with the imbalance of positive and negative cases, the difference between simulation and real cases may subtly influence the model performance. Considering to figure out the effects of balancing on modeling, we thus further undersampled the balanced training set and validated the models on testing set. The undersample training set contained 242 negative and 145 positive cases. The optimal model remained the boost tree with the accuracy and AUC value being 0.817 and 0.889 in training set, and 0.891 and 0.672 in testing set, which was slightly different with the result from non−undersampled dataset (Table and figure not shown).

4. Argument tuning

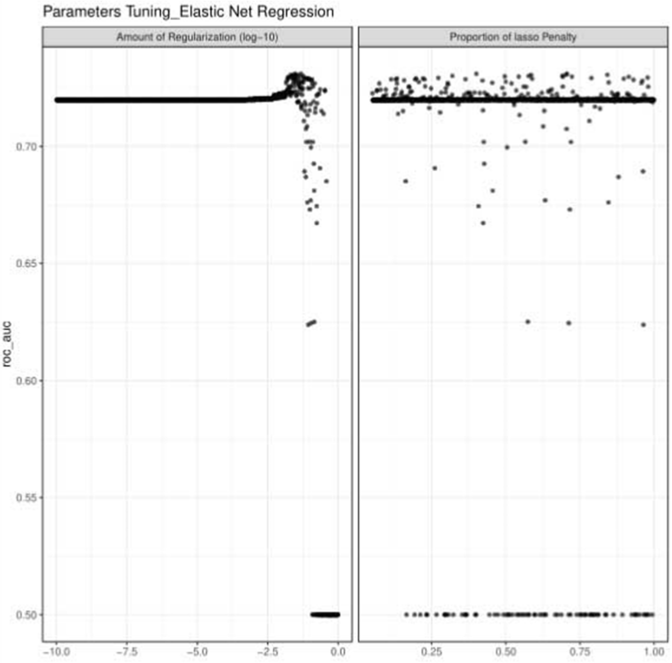

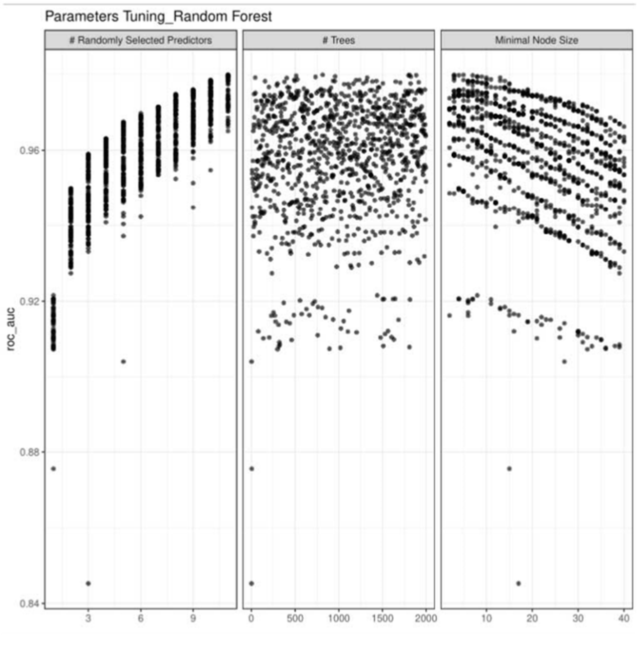

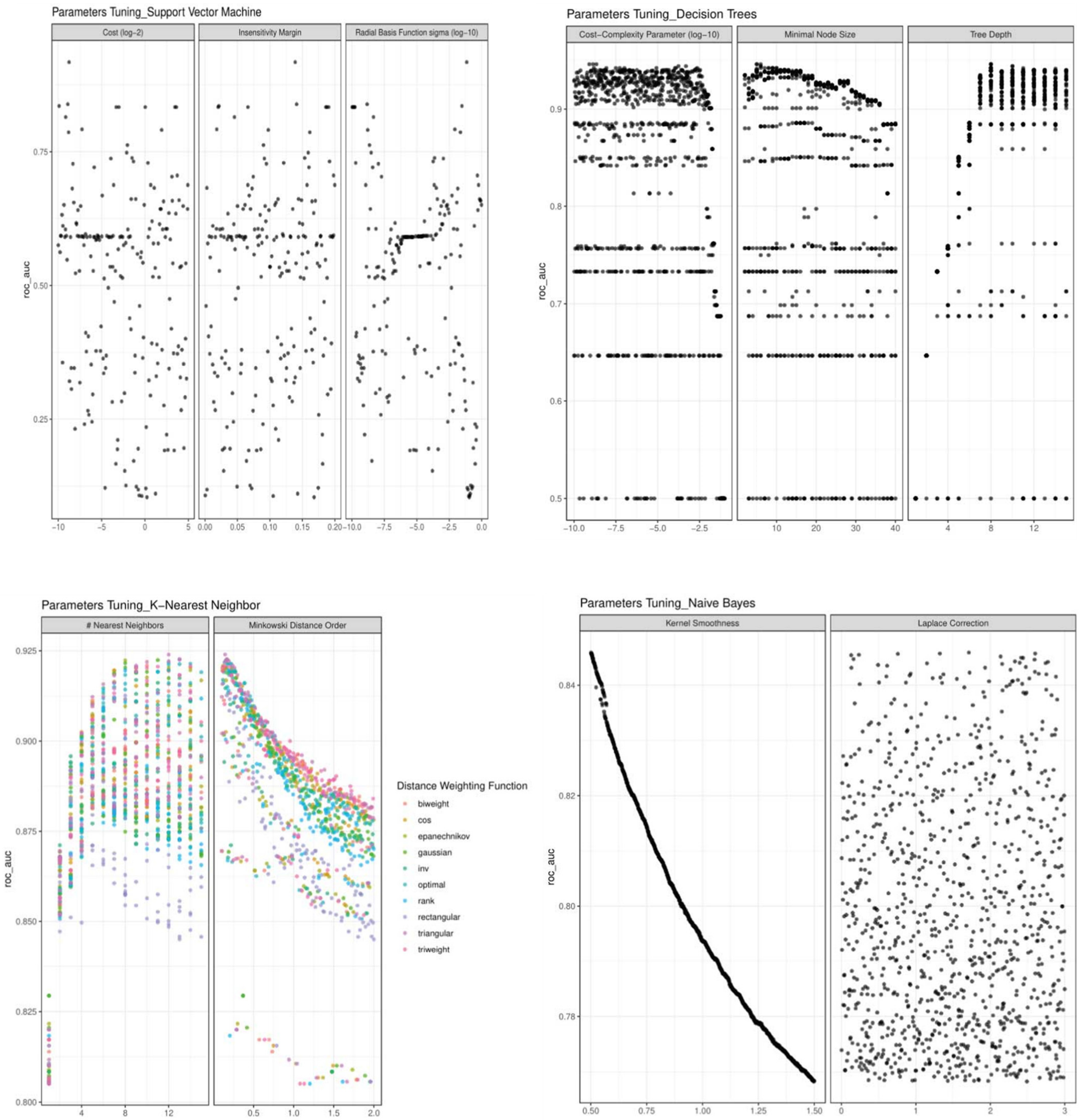
Parameters Tuning_Support Vector Machine
Cost (log-2)
Insensitivity Margin
Radial Basis Function sigma (log-10)
roc_auc
Parameters Tuning_Decision Trees
Cost-Complexity Parameter (log-10)
Minimal Node Size
Tree Depth
roc_auc
Parameters Tuning_K-Nearest Neighbor
Nearest Neighbors
Minkowski Distance Order
roc_auc
Distance Weighting Function
biweight
cos
epanechnikov
gaussian
inv
optimal
rank
rectangular
triangular
triweight
Parameters Tuning_Naive Bayes
Kernel Smoothness
Laplace Correction
roc_auc

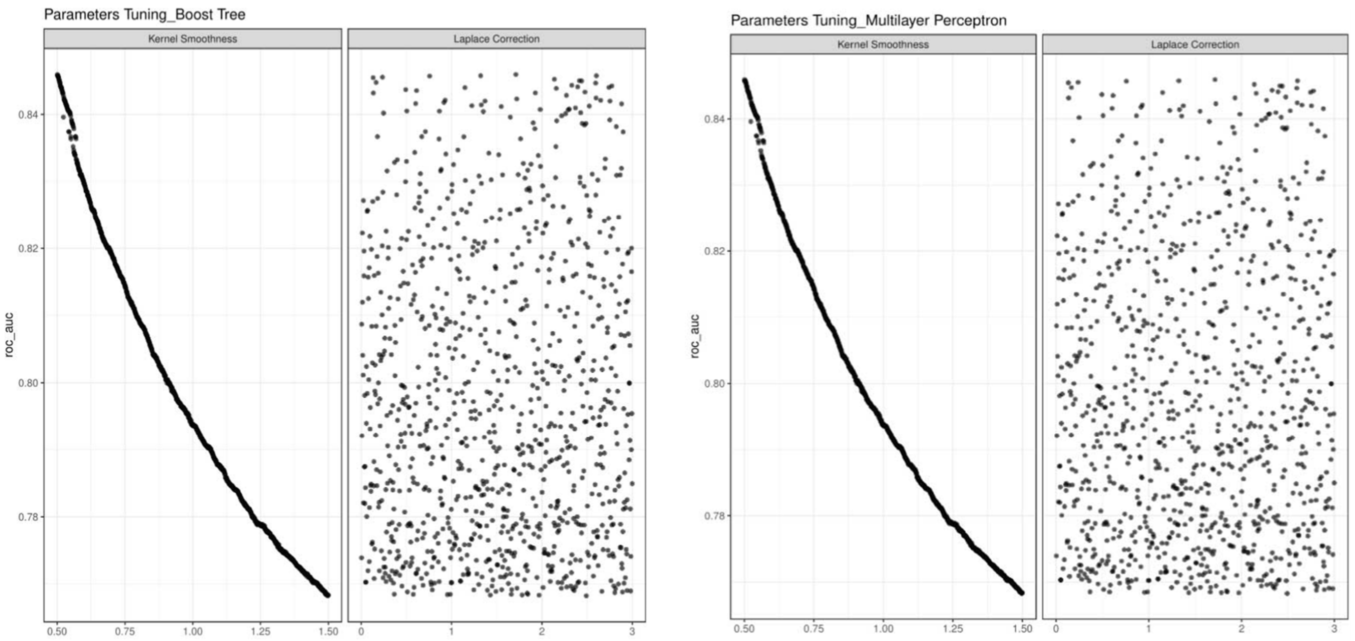

Figure S6. Argument tuning of models. We chose 8 widely used machine learning algorithms (elastic net regression, support vector machine, random forest, boost tree, decision tree, k–nearest neighbor, naïve Bayes, and multilayer perceptron) to test models in training set, along with argument tuning using the maximum entropy design with 1000 candidate values. These graphs show the grid research of the optimal combination of arguments from different algorithms. The area under the receiver operating characteristic curve (AUC) values were used to evaluate the performance of models, with the best being filtered.
